# Metabolomics Analysis Reveals that AICAR Affects Glycerolipid, Ceramide and Nucleotide Synthesis Pathways in INS-1 Cells

**DOI:** 10.1371/journal.pone.0129029

**Published:** 2015-06-24

**Authors:** Mahmoud A. ElAzzouny, Charles R. Evans, Charles F Burant, Robert T. Kennedy

**Affiliations:** 1 The Department of Chemistry, University of Michigan, Ann Arbor, United States of America; 2 The Department of Internal Medicine, University of Michigan, Ann Arbor, United States of America; 3 The Department of Pharmacology, University of Michigan, Ann Arbor, United States of America; Joslin Diabetes Center, Harvard Medical School, UNITED STATES

## Abstract

AMPK regulates many metabolic pathways including fatty acid and glucose metabolism, both of which are closely associated with insulin secretion in pancreatic β-cells. Insulin secretion is regulated by metabolic coupling factors such as ATP/ADP ratio and other metabolites generated by the metabolism of nutrients such as glucose, fatty acid and amino acids. However, the connection between AMPK activation and insulin secretion in β-cells has not yet been fully elucidated at a metabolic level. To study the effect of AMPK activation on glucose stimulated insulin secretion, we applied the pharmacological activator 5-aminoimidazole-4-carboxamide ribonucleotide (AICAR) to an INS-1 (832/13) β-cell line. We measured the change in 66 metabolites in the presence or absence of AICAR using different stable isotopic labeled nutrients to probe selected pathways. AMPK activation by AICAR increased basal insulin secretion and reduced the glucose stimulation index. Although ATP/ADP ratios were not strongly affected by AICAR, several other metabolites and pathways important for insulin secretion were affected by AICAR treatment including long-chain CoAs, malonyl-CoA, 3-hydroxy-3 methylglutaryl CoA, diacylglycerol, and farnesyl pyrophosphate. Tracer studies using ^13^C-glucose revealed lower glucose flux in the purine and pyrimidine pathway and in the glycerolipid synthesis pathway. Untargeted metabolomics revealed reduction in ceramides caused by AICAR that may explain the beneficial role of AMPK in protecting β-cells from lipotoxicity. Taken together, the results provide an overall picture of the metabolic changes associated with AICAR treatment and how it modulates insulin secretion and β-cell survival.

## Introduction

AMPK is an energy sensor that promotes metabolic changes to ensure energy balance based on nutrient availability [1]. Elevated AMP levels during starvation activates AMPK leading to stimulation of catabolic processes and inhibition of anabolic processes, whereas high glucose depletes AMP and has the opposite effects [2]. AMPK can be activated independent of nutrient level by pharmacological agents like 5-aminoimidazole-4-carboxamide ribonucleotide (AICAR), a pro-drug that is metabolized intracellularly to form the AMP analog AICAR monophosphate (ZMP). Intravenous administration of AICAR has been shown to decrease hepatic glucose output and lower blood glucose and free fatty acids in diabetic patients suggesting a potential therapeutic benefit of modulating this pathway [3].

Because AMPK affects core metabolic functions, it may be expected that its pharmacological activation may have many effects. For example, AICAR and AMPK activation may modulate glucose stimulated insulin secretion (GSIS) from β-cells in islets of Langerhans since this process is dependent on glucose metabolism to generate signals that trigger or amplify insulin secretion [4]. This potential modulation is of interest because deterioration of β-cell function represents one of the factors responsible for development of metabolic syndrome and type 2 diabetes.

The effect of AMPK activation on insulin secretion from islets and the β-cell line INS-1 has been studied [5]. AMPK over-expression in INS-1 cells significantly decreased GSIS in the presence of fatty acid. This effect was attributed to increased oxidation of fatty acids and reduction in lipid signals involved in insulin secretion [6]. AMPK activation by AICAR was also shown to potentiate insulin secretion from rat islets and INS-1 cell lines at low glucose, with no significant effect at higher glucose levels [7]; however, this effect has not been universally observed and some have reported enhancement of GSIS by AICAR [5,8], while others have shown an inhibition of GSIS [9,10]. The source of such discrepancies has not been fully investigated; however, they may result from subtle differences in conditions, such as the timing of AICAR application [5] and metabolic status of the cells used. AMPK activation by AICAR was also able to rescue INS-1 β-cells from saturated fatty acid induced toxicity by reducing lipid messengers [11]. The effects of AICAR are especially intriguing because we recently showed that the active form of AICAR, ZMP, is an endogenous metabolite that increases rapidly after glucose stimulation of INS-1 cells [4]. This effect was temporally correlated with the 2^nd^ phase of insulin secretion. AICAR added at the same time as glucose significantly increased ZMP and inhibited 2^nd^ phase insulin secretion suggesting a potential regulatory role of endogenous ZMP [4].

Studies on other tissues and cells have revealed many potential pathways that are modulated by AMPK activation and AICAR [12,13]. In adipocytes, AMPK activation inhibits hormone sensitive lipase to reduce lipolysis. In heart and macrophages, AMPK activation increases activity of phosphofructokinase B2 and B3 leading to increased glycolysis. In muscles and liver, AMPK activation inhibits glycogen synthase 1 and 2 to reduce glycogen synthesis [14]. AMPK activation also inhibits acetyl-CoA carboxylase 1 and 2 (ACC1 and ACC2), reducing fatty acid synthesis and increasing fatty acid oxidation respectively, and 3-hydroxy-3 methylglutaryl CoA reductase (HMGR), reducing cholesterol synthesis [14,15].

Although AICAR’s primary mode of action is thought to be as an AMP mimetic that causes AMPK activation, some effects of AICAR have been shown to be independent of AMPK activation. AICAR inhibited choline kinase and phosphatidyl choline synthesis in liver cells independent of AMPK [16]. AICAR has also been shown to induce apoptosis in chronic lymophcytic leukemia cells independent of AMPK leading to clinical studies of AICAR as a cancer therapeutic [17]. AMPK independent effects induced by AICAR were protective in animal models of human malignant hyperthermia from sudden death [18].

In light of the widespread use of AICAR as an activator of AMPK for research purposes, the growing interest in use of AICAR as a treatment for certain human diseases, and the potential role of AMPK in modulating insulin secretion, improved understanding of the molecular basis of its action is essential. The goal of this work was to determine the effect of AICAR on metabolism of INS-1832/13 cells. These cells were used as a model because of the direct relevance to GSIS and other processes related to type 2 diabetes such as β-cell survival. We used liquid chromatography-mass spectrometry (LC-MS) based metabolomic analysis to identify pathways affected by AICAR treatment and then used isotope labeling to track flux through selected pathways. The results reveal numerous effects of AICAR and AMPK that may be related to GSIS and β-cell survival.

## Material and Methods

### Materials

INS-1 cells [19] were kindly provided by Dr. Christopher Newgard (Sarah W. Stedman Nutrition and Metabolism Center, Duke University, Durham, NC). All chemicals were purchased form Sigma-Aldrich (St. Louis, MO) unless otherwise noted. RPMI media, fetal bovine serum, 4-(2-hydroxyethyl)-1-piperazineethanesulfonic acid (HEPES), and penicillin-streptomycin were purchased from Invitrogen Corp, (Carlsbad, CA). Anti-phospho-Acc were obtained from Cell Signaling (Massachusetts, USA). Krebs-Ringer-HEPES buffer (KRHB) was prepared containing 20 mM HEPES, 118 mM NaCl, 5.4 mM KCl, 2.4 mM CaCl_2_, 1.2 mM MgSO_4_, and 1.2 mM KH_2_PO_4_ and was adjusted to pH 7.4 with NaOH.

### Cell culture

INS-1 832/13 cells were cultured in RPMI supplemented with 2 mM glutamine, 1 mM sodium pyruvate, 10% FBS, 10 mM HEPES, 100 U/mL penicillin, 100 μg/mL streptomycin, 250 ng/mL amphotericin B, and 50 μM β-mercaptoethanol. Cells were plated at a density of ~14 x 10^3^ cells/cm^2^ and grown in 6 cm culture dishes at 37°C and 5% CO_2_ in a humidified atmosphere to ~70% confluence.

### Cell treatments

On the day of experiments, the cell culture medium was changed to KRHB containing 2 mM glucose with or without 250 μM AICAR. After 60 min incubation, 1 M ^12^C or uniformly labeled (U-^13^C) glucose was added to make a final concentration of 10 mM glucose. Cell metabolism was quenched at different time points as described in each experiment. For the dose response curve of AICAR, cells were incubated with 10 mM glucose and different concentrations of AICAR (0, 25, 125,250 and 1250 μM) for 1 h before quenching. For the experiment showing the effect of starvation on CDP-ethanolamine, cells were incubated in RPMI with 3 mM glucose or 10 mM glucose for 6 h before quenching. For DAG and ceramide analysis, the INS-1 (832/13) clonal cell line was incubated with 2 mM glucose, 250 μM AICAR and 50 μM palmitic acid for 1 h before stimulation with U-^13^C glucose for 30 minutes. In the case of CDP-ethanolamine labeling, labeled ethanolamine was also added for the 1 h pre-incubation period.

### Insulin measurement and western blots

For insulin measurements, aliquots of supernatant were diluted with 1% BSA and stored at +4°C before being assayed using a Rat/Mouse insulin ELISA Kit (Millipore, Billerica, MA). For the ACC western blot, cells were grown in 6 cm dishes to ~ 70% confluence and washed with cold PBS before the addition of 400 μl lysis buffer (RIPA buffer supplied with complete protease inhibitor cocktail and phosphatase inhibitor cocktail from Roche diagnostics). Extract was diluted with Laemmli buffer and heated to 99°C for 4 minutes (longer heating may cause ACC aggregation). Samples (30 μl) were loaded into a 12 well 5% Tris-HCl gel (Criterion gel from Bio-Rad). The electrophoresis buffer was kept ice cold during the separation process. Current was maintained stable at 0.02 ampere for 2 hours before increasing to 0.05 amp. The gel proteins were transferred to a nitrocellulose membrane overnight at 5°C. The membrane was incubated with anti-ACC antibody (Cell Signaling Technologies, part number 3662S); after developing the blot as described below it was stripped and incubated with pACC antibody (Cell Signaling Technologies, part numbers 3661S). Blots were developed with ECL (Pierce) according to manufacturer's instructions and exposed for 3 minutes or 5 seconds for total ACC and pACC respectively.

### Metabolite Measurement

Cell plates were rinsed, metabolism quenched, and metabolites extracted using a procedure described previously [4]. Briefly, cell plates were rapidly rinsed with water and quenched with liquid nitrogen. Metabolites were extracted with 8:1:1 methanol: chloroform: water and assayed by high performance liquid chromatography with time-of-flight mass spectrometry (HPLC-TOF-MS). Chromatographic separations were performed with an Agilent Technologies (Santa Clara, CA) 1200 HPLC system equipped with a Phenomenex (Torrance, CA) Luna NH2 HPLC column (2.0 mm inner bore × 150 mm long and packed with 3 μm particles) and a 2.0 × 4 mm guard column. Mobile phase A was 100% acetonitrile (ACN) and mobile phase B (MPB) was 100% 5 mM ammonium acetate adjusted to pH 9.9 with ammonium hydroxide. The gradient started at 20% MPB and ramped till 100% MPB over 20 min., was held at 100% MPB for 5 minutes, and then returned to 20% MPB for an additional 7 min. Lipids were separated on a C18 Capcell column (2 mm bore x 150 mm long packed with 3 μm particles) similar to [20]. MPA was 40% water, 40% acetonitrile and 20% methanol, and MPB was 80% isopropanol and 20% methanol. Both mobile phases contained 0.1% formic acid and 0.028% ammonium hydroxide. The gradient started with 0% MPB and linearly increased to 60% MPB over 10 min, then increased to 80% MPB over 40 min, then to 100% MPB over 5 min, was held at 100% MPB for 5 min, and then returned to 0% MPB for 10 min. Detection was performed using an Agilent Technologies 6220 LC/MSD TOF using a dual electrospray ionization (ESI) source operated in negative-ion mode for polar metabolites and both negative and positive mode for lipids.

### Data analysis and statistics

Directed analysis was performed to measure metabolites previously implicated in GSIS, e.g., glycolytic and tricarboxylic acid (TCA) cycle intermediates. Those were identified by matching retention time and accurate mass to standards as described in [4]. Undirected analysis was performed using XCMS online [21]. Features with significant changes between sample groups were putatively identified by searching m/z values against the Human Metabolome Database (http://www.hmdb.ca) and Metlin (http://metlin.scripps.edu); identifications were confirmed by analysis of authentic standards. Peak areas measured from extracted ion chromatograms of [M-H]^−^ metabolite ions with ± 70 ppm detection windows centered on the theoretical mass were used for relative quantification [4]. [M-2H]^2−^ ions were used for malonyl-CoA (mCoA) and other CoAs to improve sensitivity. Statistical comparisons were performed using unpaired Student’s t-tests comparing different time points ± AICAR. A *p* value < 0.05 was considered significant.

## Results

### AICAR effect on insulin secretion and metabolome of INS-1 cells

**Fig 1:**
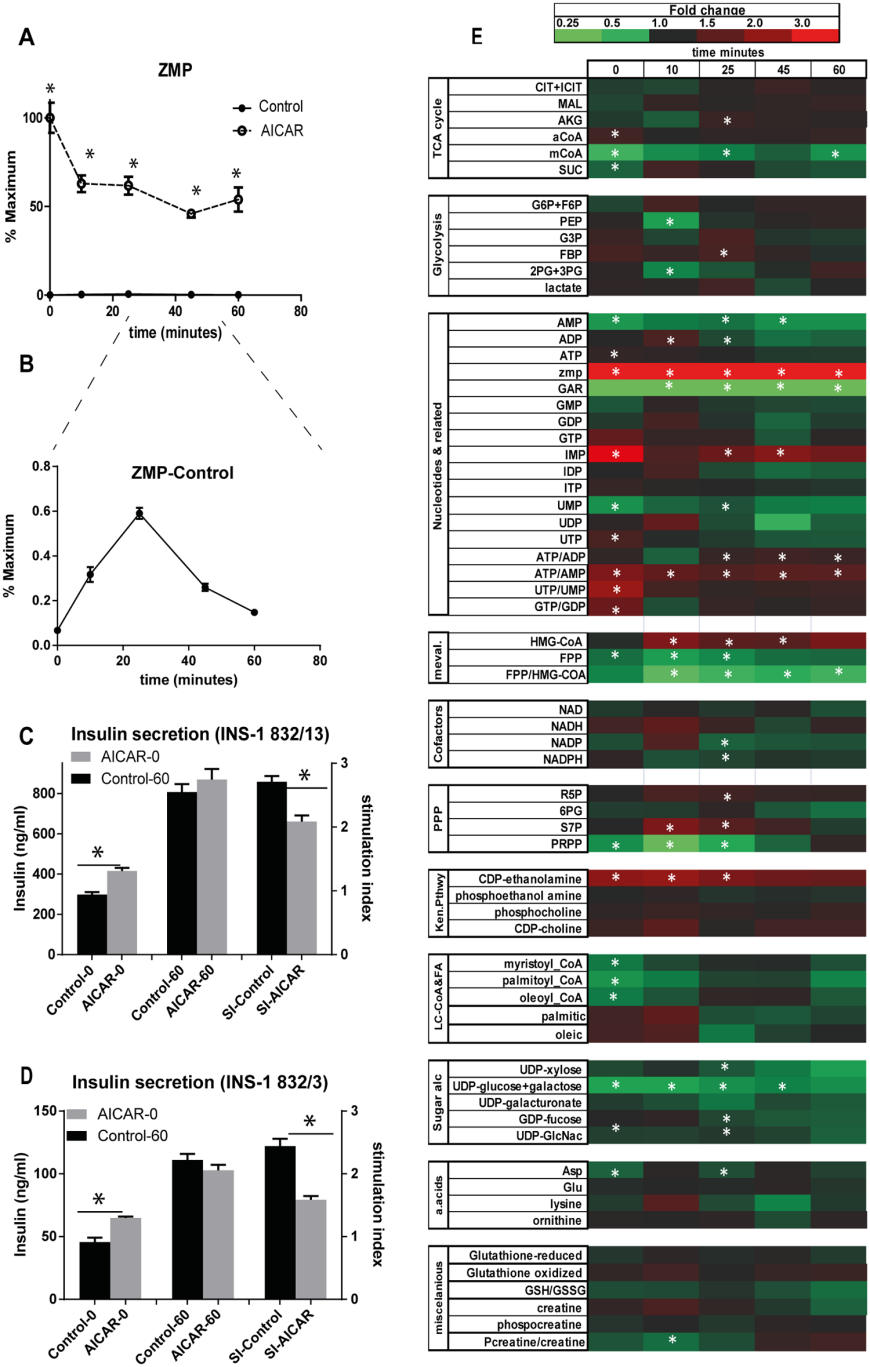
Insulin and metabolites changes with AICAR incubation. (A) ZMP levels after AICAR incubation for 1 hour followed by glucose stimulation for 60 minutes (B) zoom view of endogenous ZMP levels after glucose stimulation. (C) Insulin level and stimulation index for INS-1 cells (832/13) after AICAR incubation and after glucose stimulation for 60 minutes. (D) Insulin level and stimulation index for INS-1 cells (832/3) after AICAR incubation and after glucose stimulation for 60 minutes. (E) Heat map showing fold change of metabolites with AICAR treatment. Significantly different values are highlighted with an asterisk. Student’s t-test was performed on each time point comparing the control and AICAR treated samples, with n = 3 or 4 for each replicate.

To identify metabolic pathways that may be modulated by AICAR to alter insulin secretion, we determined its effect on the metabolome, as measured by LC-MS, in the glucose-responsive cell line INS-1. We first treated cells with 0 μM to 1250 μM AICAR for 60 min and measured ZMP. As shown in (S1) ZMP content increased linearly with AICAR concentration up to 250 μM. In subsequent studies we used 250 μM AICAR, which generated a ZMP concentration that was ~150 times greater than the highest endogenous levels (Fig 1A) and intermediate to those used in previous studies [16,22]. Although AICAR increased ZMP concentration, subsequent stimulation of INS-1 cells with 10 mM glucose decreased ZMP (p < 0.05) by nearly 50% (Fig 1A). In contrast, glucose in the absence of AICAR increased ZMP ~ 5 fold, as we have shown previously [4]; but, the resulting ZMP was still far below that generated by AICAR alone (Fig 1B). In the presence of AICAR, ZTP levels increased with time after glucose stimulation (S1 Fig)

The effect of AICAR on insulin secretion was evaluated by incubating INS-1 (832/13) cells in KRB with 2 mM glucose for 60 min in the presence or absence of 250 μM AICAR prior to treatment with 10 mM glucose. Insulin secretion was measured at low glucose (time zero) and after 60 min incubation with 10 mM glucose (Fig 1C). At 2 mM glucose, AICAR increased insulin secretion by ~33%; however, after stimulation with 10 mM glucose, insulin secretion was unaffected by AICAR, in agreement with previous studies [7]. Because of the difference in basal secretion, the stimulation index was significantly reduced in the presence of AICAR (Fig 1C). These results were reproduced in a different cell line (INS-1 832/3) (Fig 1D).

To determine the effect of AICAR on the INS-1 (832/13) cell metabolome, metabolism was quenched and metabolites extracted for LC-MS analysis at different times before and after 10 mM glucose treatment. LC-MS analysis of cell extracts allowed monitoring of 66 identified metabolites. The ratio of metabolite concentration between AICAR treated sample and control at different times during glucose treatment is summarized in Fig 1E. The zero time point illustrates the effect of 1 h incubation with 250 mM AICAR on metabolites at with INS-1 cells at 2 mM glucose. The latter time points show the effects after stepping up to 10 mM glucose. Because many metabolites change with glucose treatment, the ratio in Fig 1E actually illustrates the effect on the changes. At low glucose, AICAR decreased malonyl CoA, succinate, AMP, glycinamide ribonucleotide (GAR) and phosphoribosyl pyrophosphate (PRPP), UMP, Farnesyl pyrophosphate (FPP), long chain CoAs, UDP-N-acetylglucosamine (UDP-GlcNAC), and aspartate. AICAR increased acetyl CoA, IMP, CDP-ethanolamine, and several high energy to low energy nucleotide ratios (e.g. ATP/AMP). AICAR also modulated the changes that result during glucose that involved these same metabolites. Below we discuss those changes in more detail that may relate to insulin secretion and β-cell survival.

### AICAR decreased malonyl CoA and long-chain CoA levels

**Fig 2:**
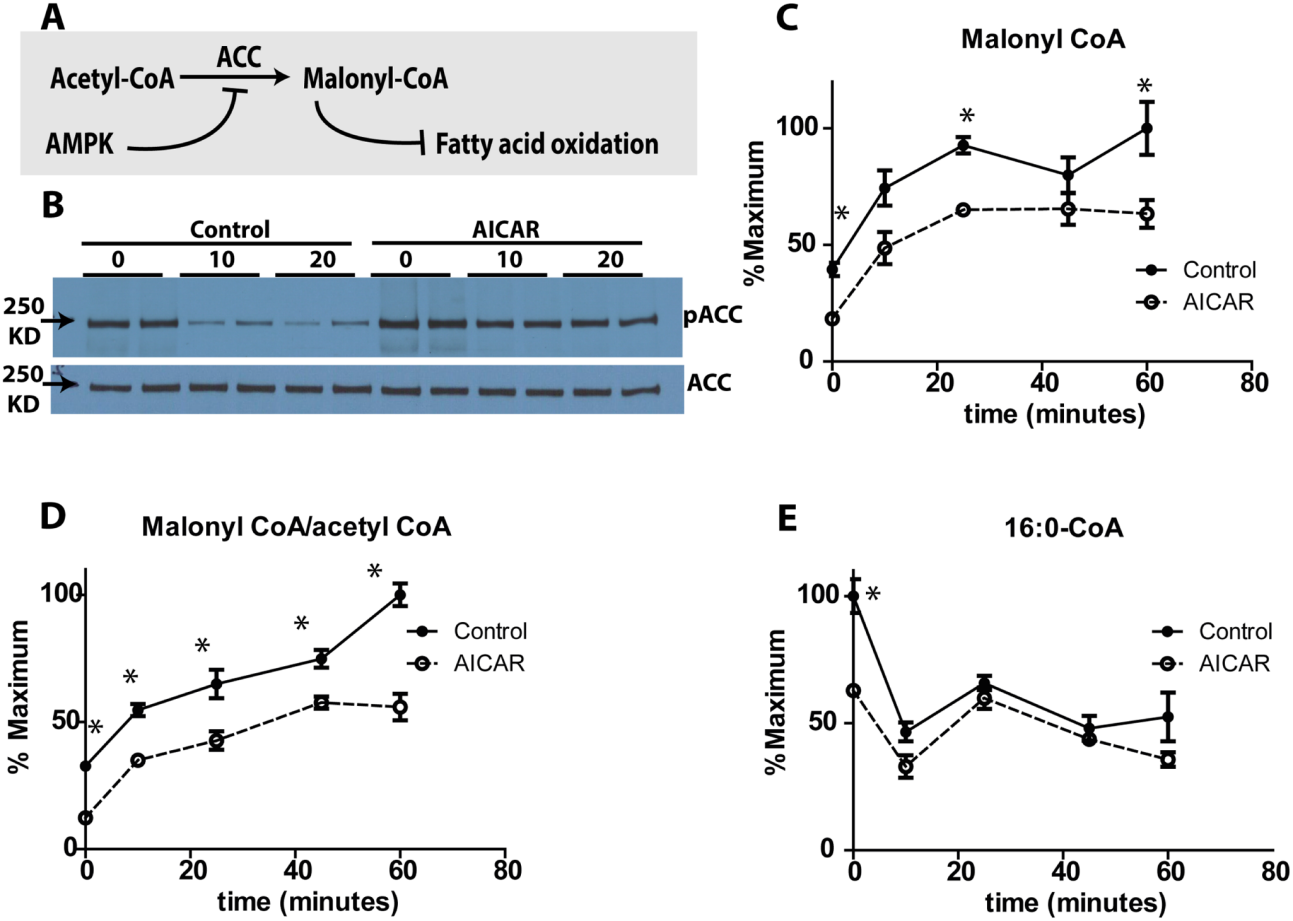
AICAR effect on Acetyl-CoA Carboxylase (ACC). (A) AMPK effect on ACC, (B) phosphoACC and total ACC after AICAR incubation and after glucose treatment for 10 and 20 minutes. Cells were incubated with/without AICAR for 1 h, followed by stimulation with 12C glucose for different time points, resulting in the illustrated levels of (C) malonyl-CoA, (D) ratio of malonyl CoA/acetyl CoA, and (E) palmitoyl-CoA. Student’s t-test was performed on all time points comparing the control and AICAR treated samples, with n = 3 or 4 for each replicate.

ZMP is known to activate AMPK which in turn phosphorylates and deactivates acetyl-CoA carboxylase (ACC) (Fig 2A). We confirmed this effect by determining ACC1 phosphorylation by Western blot analysis. In control cells, basal levels of phospho-ACC decreased significantly following addition of 10 mM glucose. AICAR treatment resulted in higher levels of basal ACC phosphorylation which decreased with glucose stimulation, but continued to be markedly higher than control cells at 20 min (Fig 2B). Total ACC was not changed by either glucose or AICAR treatment. These results mirror the changes in ZMP levels (Fig 1A). This change in AMPK activity is also reflected in the reduced levels of cellular malonyl CoA, the product of ACC. Malonyl CoA increases with glucose treatment, but the overall level is attenuated by AICAR (Fig 2C). The malonyl-CoA/acetyl-CoA ratio also increased with glucose and was offset to a lower level by AICAR (Fig 2D). The substrate of ACC, acetyl CoA, was slightly elevated at basal glucose (Fig 1E). During glucose treatment, however, acetyl CoA had small changes and after the zero time point no effect of AICAR was seen. The lack of effect on acetyl CoA may be surprising because a substrate would be expected to increase if the enzyme which consumes it is inhibited. However, previous studies have shown that acetyl CoA regulation is more complex. For example, we previously found that glucose treatment had relatively small effects on total acetyl CoA content after 10 min but treatment with ^13^C-glucose resulted in considerable ^13^C enrichment in Acetyl CoA, demonstrating substantial flux through this pathway [4]. A possible confounding factor for assessing acetyl-coA levels is that it is found in both mitochondria and cytosol. The expected accumulation of cytosolic acetyl-CoA could be masked by the mitochondrial pool. Malonyl-CoA, which is only cytosolic, can be probed more easily.

Malonyl CoA inhibits CPT-1 and fatty acid oxidation with a consequence of increasing availability of long-chain CoA in cytosol. As expected, AICAR reduced long-chain CoAs at low glucose (time 0 in Fig 2E). Interestingly, glucose treatment without AICAR also decreases long-chain CoAs. We have recently shown that this glucose-stimulated decrease in long-chain CoA is due to consumption by rapid esterification with glycerol-3-phosphate, which dramatically increases with glucose treatment, to produce glycerolipids [4,23]. While AICAR reduces long-chain CoAs at low glucose (Fig 2E, time zero), its effect is not additive with glucose so that at high glucose AICAR has no effect on long-chain CoAs (Fig 2E).

### AICAR increased HMG-CoA and decreased farnesyl pyrophosphate

**Fig 3:**
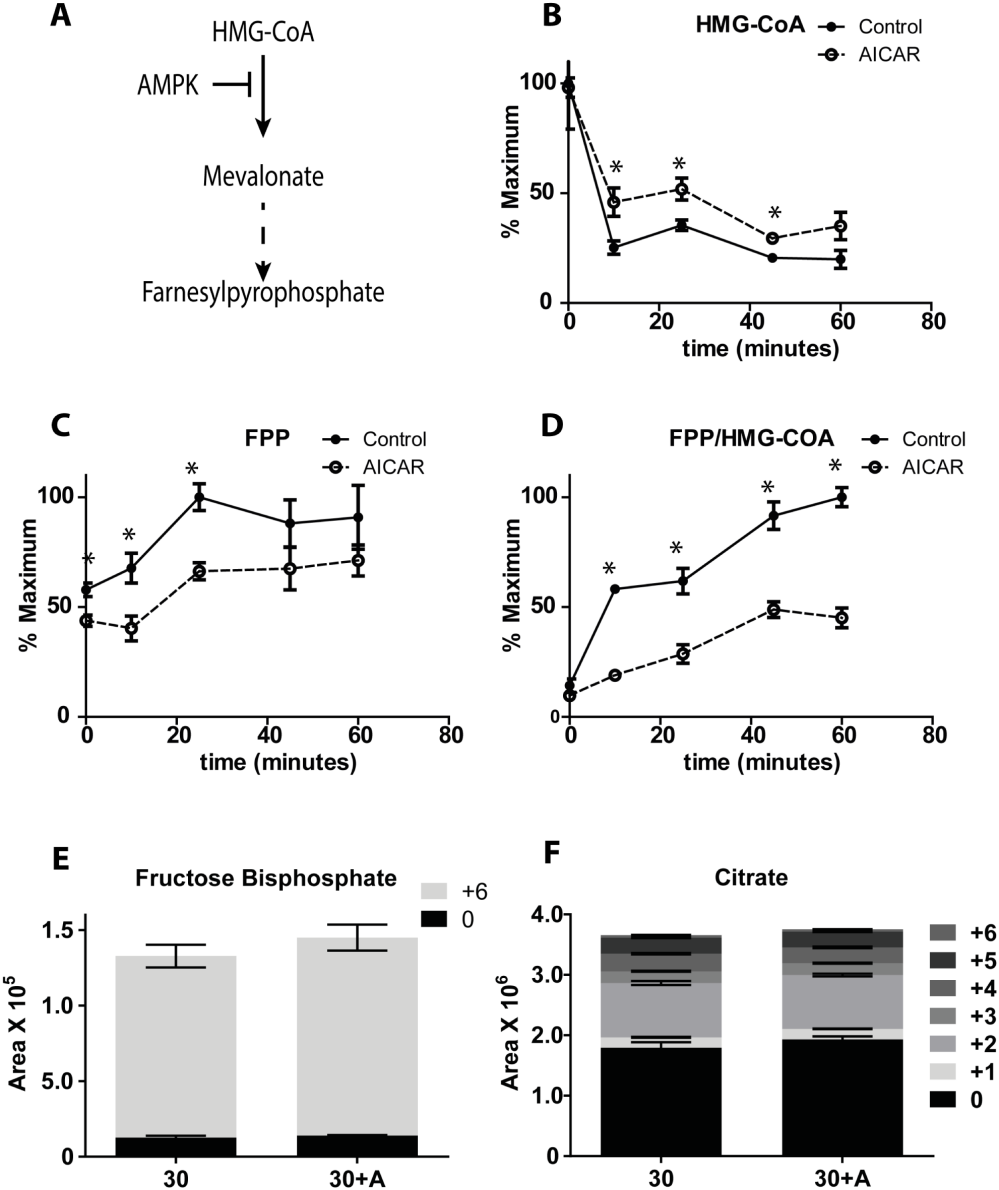
AICAR effect on HMGR and Central Carbon metabolism. (A) AMPK effect on mevalonate pathway. Cells were incubated with/without AICAR for 1 h, followed by stimulation with 12C glucose for different time points, resulting in the illustrated levels of (B) HMG-CoA, (C) farnesyl pyrophosphate and (D) ratio of farnesyl pyrophophate/ HMG-CoA. Cells were incubated with/without AICAR for 1 h, followed by stimulation with U-^13^C glucose for different time points, resulting in the illustrated levels of different isotopomers of (E) fructose bisphosphate and (F) Citrate. Student’s t-test was performed on all time points comparing the control and AICAR treated samples, with n = 3 or 4 for each replicate.

AMPK also phosphorylates and deactivates HMG-CoA reductase (Fig 3A) [14]. The substrate of HMG-CoA reductase, HMG-CoA, decreases ~ 4 fold with glucose treatment (Fig 3B). AICAR did not affect the initial concentration but did blunt the decrease evoked by glucose, consistent with the deactivation of HMG-CoA reductase (Fig 3A). Farnesyl pyrophosphate (FPP), a downstream metabolite of HMG-CoA that was detected in the metabolomic analysis, was also not strongly affected at low glucose, but its increase induced by glucose was blunted by AICAR (Fig 3C). The combined effects, plotted as the ratio of FPP/HMG-CoA (Fig 3D), suggest that AICAR deactivation of HMG-CoA reductase lowers flux through this pathway to reduce net concentration of downstream products even with elevated glucose. Since a similar pathway is involved in cholesterol synthesis, this result agrees with the AICAR induced reduction of cholesterol accumulation in myotubes [24] and macrophages [25].

### AICAR did not affect glycolysis or TCA metabolites

Glucose stimulation is known to increase flux into glycolysis and the TCA cycle yielding an increase in ATP/ADP ratio in β-cells [26]. Key metabolites in these pathways, such as glucose-6 phosphate, fructose-bisphosphate and citrate, were not affected by AICAR (Fig 1E). To determine if flux through glycolysis and the TCA cycle was affected, cells were treated with U-^13^C glucose for 30 min and the resulting ^13^C- labeling of fructose bisphosphate and citrate was measured. No significant effects of AICAR were detected (Fig 3E and 3F) suggesting little effect of AICAR on central metabolism. This result was consistent with the lack of effect on the ratio of ATP/ADP at early time points (Fig 1). AICAR did slightly increase ATP/ADP ratio after 25 minutes of glucose treatment; however, this effect was mainly the result of a decrease in ADP concentration (described below).

### AICAR affects downstream of pentose phosphate pathway

**Fig 4:**
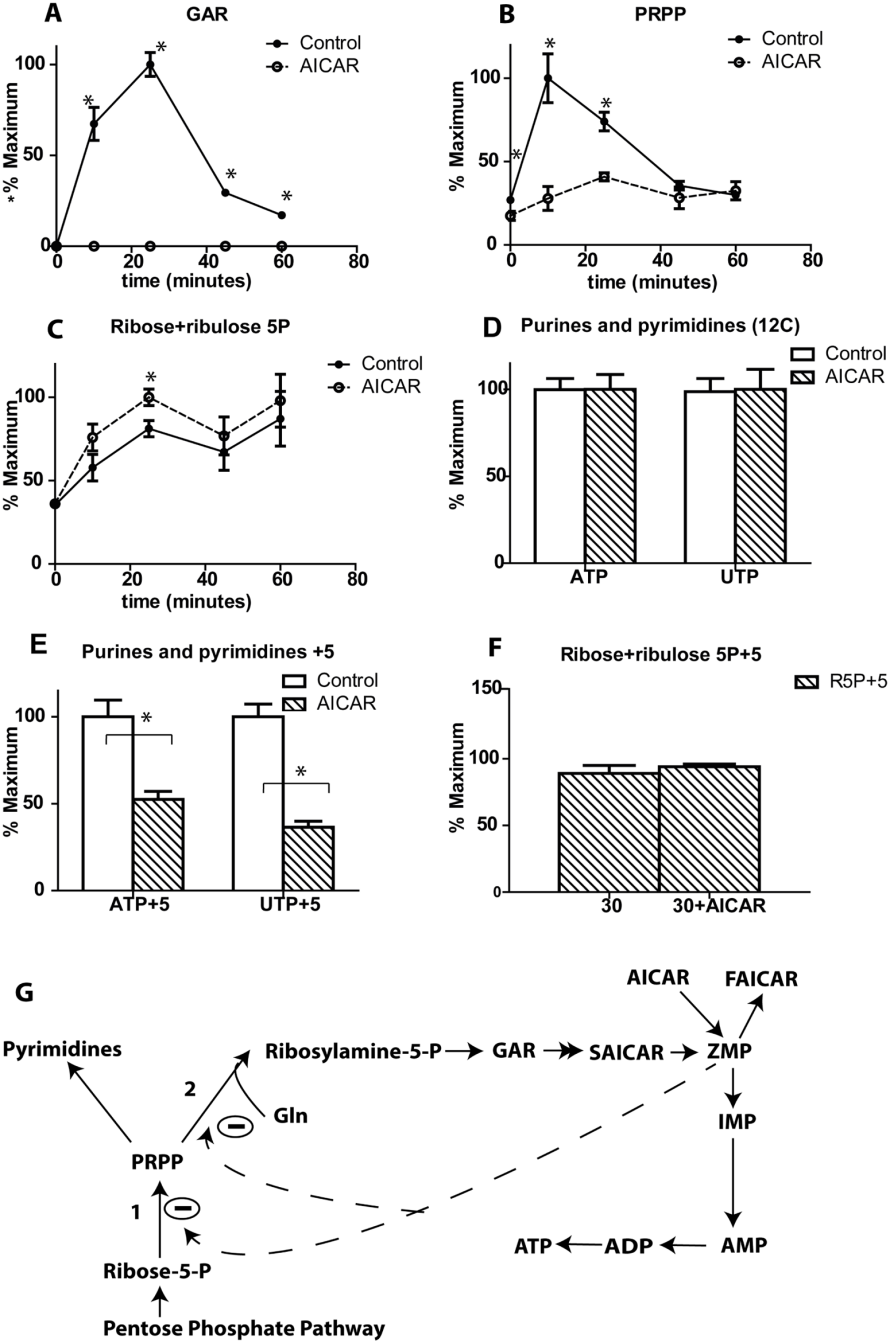
Effect of AICAR on pentose phosphate and purine pathway metabolites. Cells were incubated with/without AICAR for 1 h, followed by stimulation with 12C glucose for different time points, resulting in the illustrated levels of (A) GAR, (B) PRPP and (C) Ribose and ribulose-5-P. Cells were incubated with/without AICAR for 1 h, followed by stimulation with U-^13^C glucose for different time points, resulting in the illustrated levels of D) Unlabeled ATP and UTP, (E) +5 labeled ATP and UTP, and (F) +5 labeled ribose+ribulose-5 phosphate. The percentage of maximum was calculated based on the maximum of each species. (G) The purine and pyrimidine pathway (1) PRPP synthase (2) PRPP amidotransferase. Student’s t-test was performed on all time points comparing the control and AICAR treated samples, with n = 3 or 4 for each replicate.

Our metabolomic analysis showed that glucose significantly increased levels of glycinamide ribonucleotide (GAR) and phosphoribosyl pyrophosphate (PRPP), but that AICAR substantially blunted this increase (Figs 1E, 4A and 4B). PRPP is a metabolite that links the pentose phosphate pathway with the purine and pyrimidine synthesis pathway (Fig 4G). GAR is a metabolite in the early steps of the purine synthesis pathway (Fig 4G). Thus these results suggest that glucose normally activates the purine synthesis pathway in INS-1 cells, but this effect is blocked by AICAR. In accordance with this finding, the substrate pentose phosphates increased slightly with AICAR incubation (Figs 1 and 4C).

To further understand the effect of AICAR on GAR and PRPP concentration, we used U-^13^C labeled glucose to monitor the flux of glucose into the purine and pyrimidine pathway. Using U-^13^C labeled glucose, newly synthesized ATP and UTP could be measured by LC-MS by observing the 5 Da mass increases caused by addition of a ^13^C_5_-labeled ribose sugar to adenine or uridine. The levels of unlabeled ATP or UTP did not change (Fig 4D), while the levels of the 5 labeled ATP or UTP decreased significantly in the presence of AICAR (Fig 4E) indicating that glucose flux into the purine and pyrimidine pathway is reduced. The labeling of pentose phosphate metabolites did not show significant differences (Fig 4F).

### AICAR affected Kennedy pathway of phosphatidyl ethanolamine synthesis, decreased flux of glucose in glycerolipids pathway, and decreased ceramides

**Fig 5:**
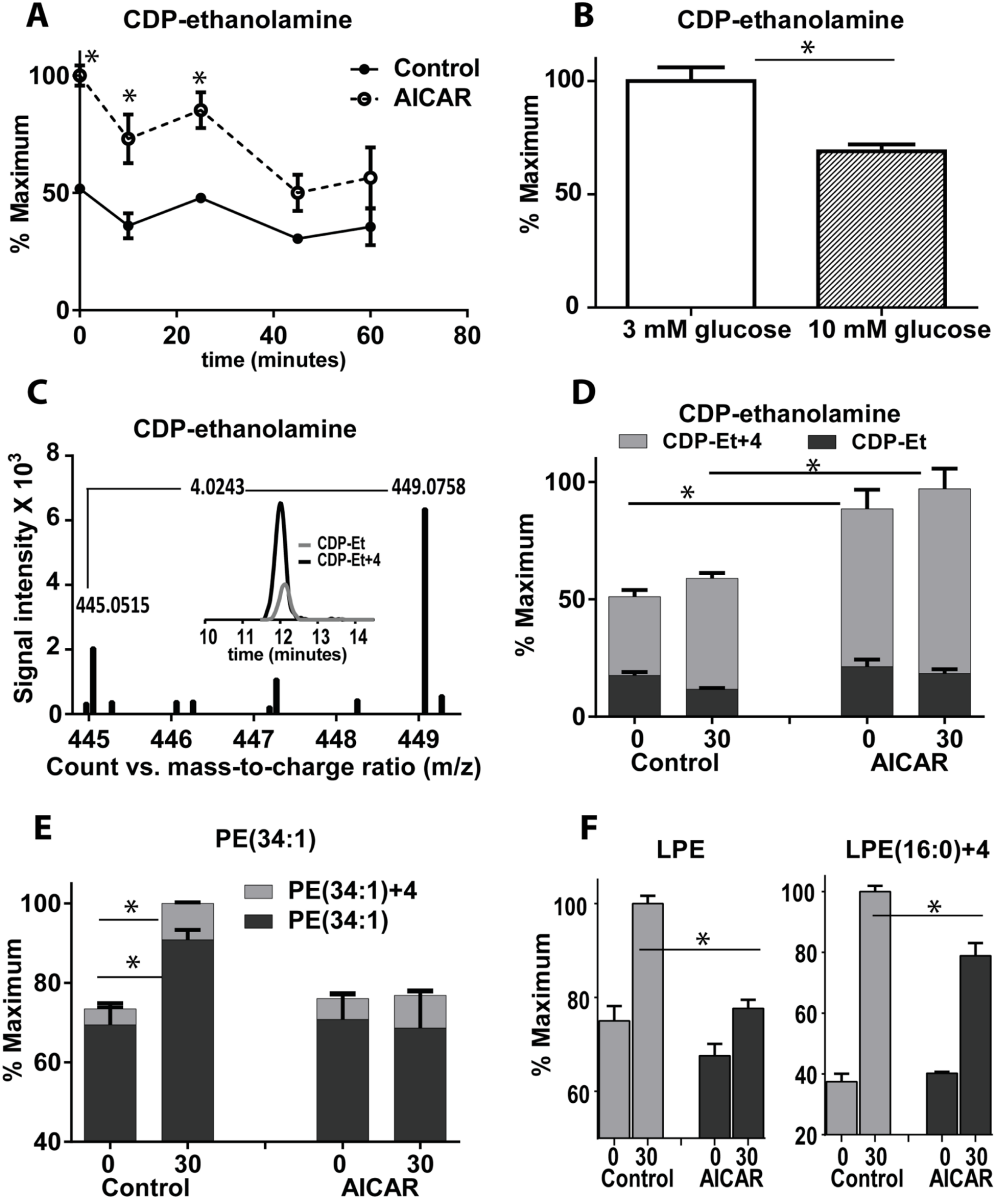
Effect of AICAR on metabolites in the Kennedy pathway for PE. (A) CDP-ethanolamine after incubation with/without AICAR for 1 h followed by stimulation with glucose for different time points (B) CDP-ethanolamine levels after starvation for 6 h at low glucose (C) Extracted ion chromatogram and mass spectrum of labeled and unlabeled CDP-ethanolamine-after incubation with +4 ethanolamine for 1 h before glucose treatment for 30 minutes (D) levels of labeled and unlabeled CDP-ethanolamine-after incubation with +4 ethanolamine for 1 h and stimulation with glucose for 30 minutes. (E, F) Levels of PE and LPE after incubation for 1 hour with +4 ethanolamine and stimulation with 12C glucose for 30 minutes. Student’s t-test was performed with n = 3 or 4 for each replicate.

Untargeted metabolomic analysis of polar metabolites revealed an increase in CDP-ethanolamine with AICAR incubation (Figs 1 and 5A and S1 Fig), in agreement with previous data showing an increase of CDP-ethanolamine in hepatocytes with AICAR treatment [22]. Low glucose treatment for 6 h (Fig 5B) also increased CDP-ethanolamine, which suggests that the CDP-ethanolamine increase seen with AICAR is AMPK dependent.

According to the Kyoto Encyclopedia of Genes and Genomes (KEGG) [27,28], CDP-ethanolamine is used only in de novo phospholipid synthesis (Kennedy pathway). To confirm the peak assignment (no authentic standard is available for this metabolite), we incubated cells with 4-^2^H labeled ethanolamine. A compound with mass of 449.0758, consistent with the predicted mass of M+4 CDP-ethanolamine, was formed which co-eluted with the unlabeled form, confirming correct peak assignment (Fig 5C). Comparable results were obtained with 2-^13^C-labeled ethanolamine (S2 Fig). Although a slight shift in retention time was seen with the deuterated metabolite, as expected [29], it provided a higher mass shift of +4 Da compared to 2-^13^C analog. The higher mass shift was useful to monitor flux changes induced by AICAR in the Kennedy pathway for phosphatidylethanolmine (PE) synthesis.

To monitor the effect of AICAR on the PE synthesis pathway, we incubated cells with 4-^2^H labeled ethanolamine and 50 μM palmitic acid for 1 h before stimulation with ^12^C glucose. AICAR caused an accumulation of labeled CDP-ethanolamine (Fig 5D). Glucose stimulation increased both labeled and unlabeled PE (34:1) levels, while AICAR blunted this increase (Fig 5E). These results suggest that AICAR reduced the effect of glucose on glycerophospholipid synthesis. To confirm that the blunted increase in PE is not due to increased lipolysis, we measured the levels of lysophosphatidylethanolamine (LPE) which is generated from phospholipase A activity. In control cells, glucose treatment increased labeled and unlabeled LPE (16:0), while AICAR blunted this increase (Fig 5F).

**Fig 6:**
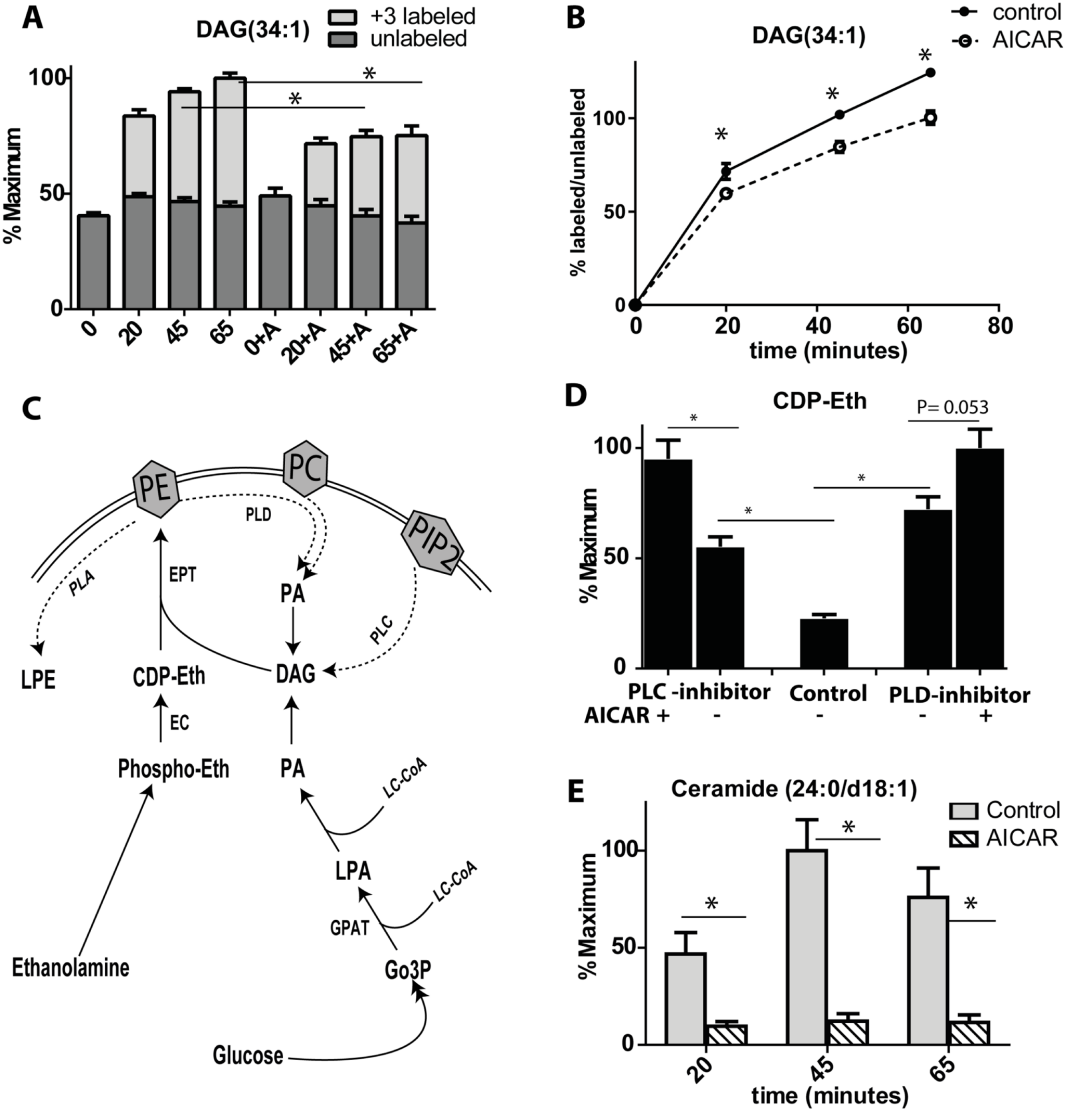
Effect of AICAR on Ceramides and glycerolipids synthesis pathway. (A) levels of DAG (34:1) isotopomers after stimulation with U-^13^C glucose for 65 minutes. (B) ratio of +3 labeled DAG/unlabeled DAG (C) Glycerolipid and Kennedy pathway for PE synthesis: ECT-ethanolamine phosphate cytidylyltransferase, EPT-ethanolamine phosphotransferase, GPAT-glycerol-3-phosphate O-acyltransferase, (D) CDP-ethanolamine levels after incubation of cells at 2 mM glucose for 60 minutes +/- AICAR +/- phospholipase C (PLC) inhibitor (U-73122) 20 μM or +/- phospholipase D inhibitor Cay10593 (60 μM). (E) Ceramide levels after incubation of cells with 50 μM palmitic acid +/- AICAR 250 μM for 1 h before stimulation with U-^13^C glucose for different time points. Student’s t-test was performed with n = 3 or 4 for each replicate.

To further understand why AICAR reduces the effect of glucose on lipogenesis and lipolysis, we monitored glucose flux into the glycerolipid pathway using U-^13^C glucose. Cells were incubated cells with 50 μM palmitic acid for 1 h followed by addition of U-^13^C glucose to allow lipogenesis and formation of ^13^C-labeled glycerolipids (M+3 due to ^13^C-glycerol-phosphate backbone). By observing the absolute amount (Fig 6A) as well as the labeling ratio (M+3 labeled/unlabeled glycerolipids) with time (Fig 6B) it is evident that AICAR induced a consistent decrease of glucose flux into the glycerolipid pathway. This decrease in flux was coupled with an accumulation of glycerol-3-phosphate (S3 Fig) which may be due to AICAR inhibiting GPAT [30].

In the Kennedy pathway, CDP-ethanolamine is consumed by condensation with diacylglycerol (DAG) to form PE (Fig 6C). The increase in CDP-ethanolamine induced by AICAR could be secondary to the reduced lipogenesis and the reduction of DAG. DAG can be formed also through lipolysis of membrane lipids by phospholipase C and D (Fig 6C). To determine if CDP-ethanolamine levels would increase if lipolysis was inhibited, we used inhibitors of phospholipase C (U-73122) or D (Cay10593) [31,32]) and determined the levels of CDP-ethanolamine. We found that both phospholipase inhibitors cause a significant increase in CDP-ethanolamine which was further augmented by the addition of AICAR (Fig 5D), suggesting a marked reduction in the consumption of CDP-ethanolamine.

To detect other possible effects of AICAR on lipids we performed untargeted metabolomic profiling on INS-1 cells following 1 h treatment with 50 μM palmitic acid and 250 μM AICAR followed by 1 h stimulation with 10 mM U-^13^C glucose. The most prominent identified changes were in ceramides (identification confirmed by matching accurate mass and retention time with external standard), which decreased significantly with AICAR treatment (Fig 6E). We also found that concentrations of serine were reduced in a dose-dependent manner after the addition of AICAR (S4 Fig) consistent with the previously described inhibition of serine palmitoyltransferase II [33].

## Discussion

GSIS is blunted in the presence of AICAR and a variety of reasons for this effect have been proposed [9,34]. We used LC-MS based metabolomics to probe potential metabolic effects of AICAR in the β-cell line INS-1. We found that AICAR treatment affected several metabolic pathways through both AMPK-dependent and independent processes. These pathways appear to be relevant to the β-cell secretory function as well as the transformed nature of the cell line. These pathways might represent novel targets for future development of treatments for diabetes and cancer.

### Effect of AICAR on GSIS related pathways

Metabolomics allows us to evaluate the effect of AICAR on several key metabolites involved in GSIS. The increase in ATP/ADP ratio within the first few minutes of glucose stimulation is known to trigger first phase insulin secretion [4,26]. AICAR did not affect ATP/ADP ratio at early time points, but evoked a slight increase after 25 minutes. This increase in ATP/ADP ratio is due to a decrease in ADP concentration, perhaps due to ZMP induced inhibition of purine biosynthesis (Figs 1 and 5).

AICAR treatment reduced the levels of long-chain CoAs which are known to have several roles in GSIS. Long-chain CoAs can potentiate opening K_ATP_ channels, counteracting the closure that occurs with increases in ATP/ADP ratios so that the reduction in long-chain CoAs may be stimulatory to GSIS [35]. We [4,23] and others [36] have found that glucose lowers the long-chain CoA concentration suggesting a possible contributing factor to the “triggering” of GSIS [37]. AICAR lowered the long-chain CoA concentration at low glucose concentrations, but did not significantly lower it further with glucose stimulation. These results might explain the elevated insulin secretion at low glucose shown with AICAR incubation.

The succinate pathway hypothesis [38] suggests that part of the ability of glucose to enhance insulin secretion involves the formation of HMG-CoA through HMG-CoA reductase. Supporting the potential importance of this pathway, we have previously shown that glucose increases flux through HMG-CoA reductase yielding a net decrease in HMG-CoA and an increase in FPP, a downstream metabolite [4], an observation reproduced here. FPP is involved in prenylation of proteins, a modification that may promote exocytosis [39]. The inhibition of HMG-CoA reductase by statin drugs inhibits GSIS [38]. In this study, AICAR blunted the effect of glucose on this pathway resulting in higher HMG-CoA and lower FPP. Such results are consistent with inactivation of HMG-CoA reductase by AICAR through AMPK, and could explain some of AICAR’s inhibitory effects on GSIS.

Glycerolipid synthesis is believed to be involved in promoting and sustaining GSIS [40]. Lipid metabolites, such as DAG may serve as proximal metabolites in exocytosis while pathways supporting production of these compounds are critical for providing a supply at the proper time for GSIS. AICAR evoked a number of complex changes in lipid metabolites, including a decrease in DAG that may play a role in its net inhibitory effect on GSIS. Our data suggest that the reduced DAG is due to a decrease in glucose flux into the glycerolipids cycle. Using U-^13^C glucose, we showed that the formation of +3 labeled DAG was reduced in the presence of AICAR, suggesting less esterification with glucose-derived glycerol-3-phosphate. Although AICAR did not cause accumulation of glycerol-3-phosphate during glucose treatment (Fig 1E), it did cause a near doubling of glycerol-3-phosphate with glucose in presence of 50 μM palmitic acid (S3 Fig) a condition where flux into glycerolipids is high [23]. Similar accumulation of glycerol-3-phosphate was seen previously in myeloma cells with AICAR treatment [41]. Together the data suggest that the decrease in DAG is likely due to reduced glycerol-3-phosphate and long-chain CoA esterification by GPAT, consistent with AMPK inhibition of GPAT [30]. Inhibition of GPAT would also explain the increase in CDP-ethanolamine found with AICAR. This effect may explain the reduction of fatty acid potentiation of insulin secretion in INS-1 cells that over-expressed AMPK [6].

### ZMP effect on survival pathways

Our results point to several ways that AICAR may affect survival or growth of cells. AICAR decreased the de novo synthesis of purine and pyrimidine metabolites by decreasing glucose flux through their biosynthetic pathways. Small, but significant accumulation of pentose phosphate metabolites was seen following AICAR treatment, which would suggest that AICAR caused inhibition of enzymes that link the pentose phosphate pathway with the purine and pyrimidine pathway. Potential sites of action are PRPP synthase, the enzyme responsible of formation of PRPP, and PRPP amido transferase, which catalyzes the conversion of PRPP and glutamine to ribosyl amine-5 phosphate which forms GAR (Fig 4G). Interestingly, both enzymes are known to be inhibited by metabolites in the purine synthesis pathway, including AMP [42,43]. The inhibition seen with AICAR may be mediated by the AMP analogue, ZMP.

These results bolster the idea that AICAR may be a potential anti-metabolite therapy for cancer treatment. It was shown recently that AICAR induced apoptosis in cancer cells independent of AMPK activation [17,41]. The cytotoxic effect [41] was suggested to be due to inhibition of enzymes in the pyrimidine pathway, mainly UMP synthase, which would agree with other published data [44]. Although the metabolite measurements were made at 8 h compared to our 1 h, they also observed a decrease in PRPP.

Another metabolic change which may be relevant to β-cell health is the significant decrease in ceramide levels seen with AICAR incubation. Ceramides are suggested to be a lipid second messenger responsible for β-cell death after its exposure to saturated fatty acid [45]. Another study showed that AMPK activation by AICAR inhibited serine palmitoyl transferase II (SPT II) transcription and lowered palmitate-induced ceramide formation in skeletal muscle [33]. Because we found two precursors of ceramide, serine and palmitoly CoA, to be reduced by AICAR, the large decrease in ceramide formation may be due to reduced substrate availability. It was shown previously that AICAR reduced palmitic acid induced apoptosis in INS-1 cells [11]. This was attributed to reduced glycerolipids synthesis and reduced glucolipotoxicity. Our data support this finding and suggest that this beneficial effect of AICAR could be attributed to the reduction of glycerolipids synthesis as well as the reduction of ceramides.

### CDP-Ethanolamine as a probe for glycerolipids cycle activity

During the course of these studies, we found that AICAR treatment resulted in the accumulation of CDP-ethanolamine, likely playing a role in the reduction of the formation of PE. While no direct role has been uncovered for PE in insulin secretion, we have recently found that inhibition of the GPR40 receptor, a therapeutically relevant target for the treatment of diabetes [46], results in the accumulation of CDP-ethanolamine, concomitant with reduction in lipid cycling in β-cells [23]. While CDP-ethanolamine may not be a direct mediator of insulin secretion, this readily detectable metabolite appears to serve as a simple probe for activity of the glycerolipids cycle.

### Endogenous ZMP Compared to AICAR-derived ZMP

AICAR results in high ZMP concentrations in the cell, but glucose itself increases ZMP, likely through de novo synthesis [4,47]. Although glucose results in an increase in ZMP in the absence of AICAR, it begins to decline after 25 min, perhaps due to the inhibition of its own synthesis as suggested above and supported by the simultaneous decline of its precursor GAR (Fig 4A). It has previously been suggested that endogenous ZMP might act to restrain GSIS during second phase [4]. ZMP produced by glucose does not result in an increase in ACC activation that is detectable by Western blot [4] and therefore it does not seem to overcome the effect of decreased AMP in presence of glucose. Thus, any signaling effects of ZMP derived from glucose are likely to act through non-AMPK mechanisms, some of which were delineated here. In the presence of AICAR, ZMP is much higher (150-fold) but glucose appears to stimulate a slow decline (Fig 1A). It is unlikely that this decline is due to the same reason as that seen at 25 min for glucose-derived ZMP. We speculate that the fall could relate to reduced AICAR transport into cells or increased breakdown of ZMP through a variety of pathways [48] or increased phosphorylation of ZMP to ZDP and ZTP. However, the latter can only account for a small amount of the decline as relative levels of ZMP were ~100 fold higher than ZTP (S1 Fig)

## Conclusion

Metabolomic analysis revealed alterations in numerous biochemical pathways in INS-1 cells induced by AICAR to provide insight into the role of AICAR and AMPK in insulin secretion. AICAR did not alter ATP/ADP ratio, a key trigger for glucose-stimulated insulin secretion; however, it did affect the intracellular of concentration several known or putative modulators of insulin secretion including long-chain CoAs, malonyl CoA, DAG, HMG-CoA, and FPP in ways that would be consistent with elevated basal secretion or inhibited stimulated secretion. AICAR also altered pathways involved in cell growth or survival including a decrease flux in the purine and pyrimidine pathways and a decrease accumulation of ceramides. This latter effect might help to explain the protective role of AMPK in decreasing fatty acid induced toxicity in β-cells. The results also show that metabolomics analysis provides an effective way to characterize effects of drugs on multiple pathways.

## Supporting Information

S1 Fig (A) Levels of ZMP after increasing dose of AICAR. (B) Time course of ZTP and ZMP levels after glucose stimulation (C) levels of CDP-ethanolamine after increasing dose of AICAR.
(TIF)

S2 Fig Chromatogram and mass spectra of 2-^13^C labeled CDP-ethanolamine. (TIF)

S3 Fig AICAR effect on the levels of G3P. INS-1 cells was incubated with 50 μM Palmitic acid, 2 mM glucose and ethanolamine +/- 250 μM AICAR for 1 h.
(TIF)

S4 Fig AICAR Dose response curve. Levels of serine after increasing dose of AICAR
(TIF)
