# Supplementary figures and images for: Metabolomics Analysis Reveals that AICAR Affects Glycerolipid, Ceramide and Nucleotide Synthesis Pathways in INS-1 Cells
Source: PLoS One. 2015 Jun 24;10(6):e0129029. doi: 10.1371/journal.pone.0129029 (PMC4480354; doi:10.1371/journal.pone.0129029)

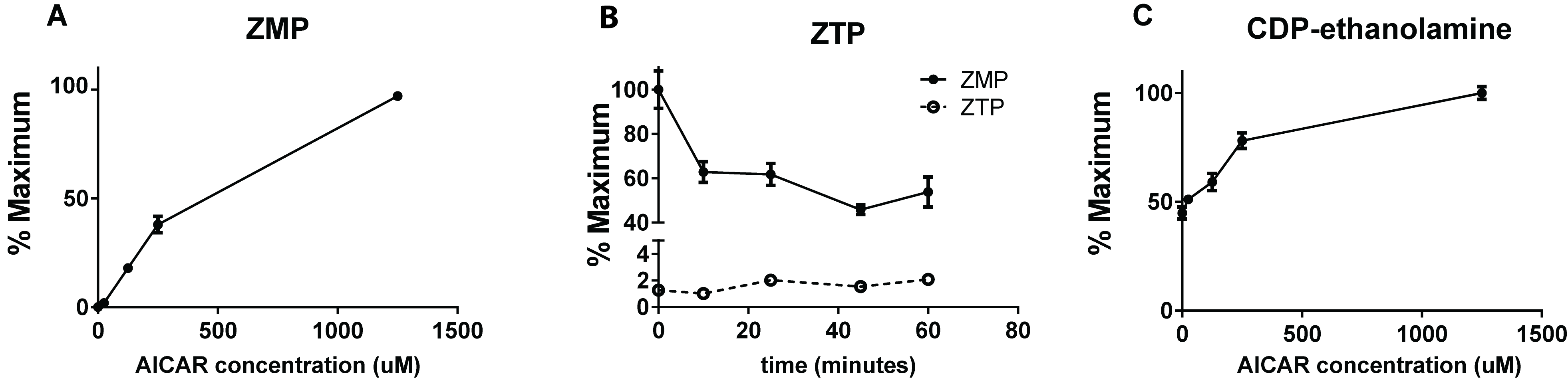

Supplement: S1 Fig — (A) Levels of ZMP after increasing dose of AICAR. (B) Time course of ZTP and ZMP levels after glucose stimulation (C) levels of CDP-ethanolamine after increasing dose of AICAR. (TIF) [file pone.0129029.s001.tif]

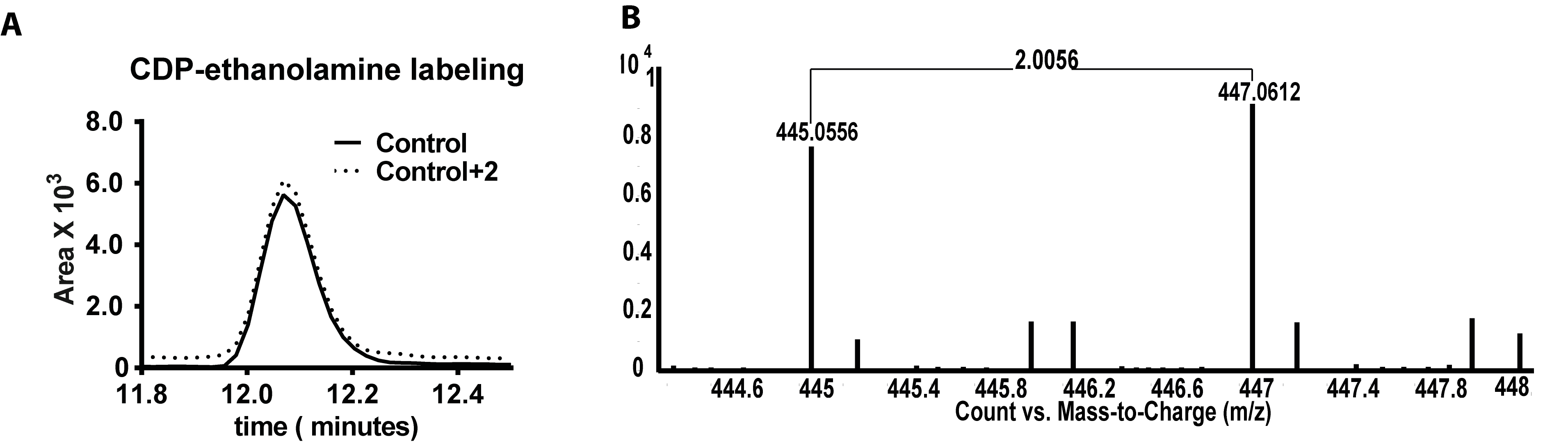

Supplement: S2 Fig — (TIF) [file pone.0129029.s002.tif]

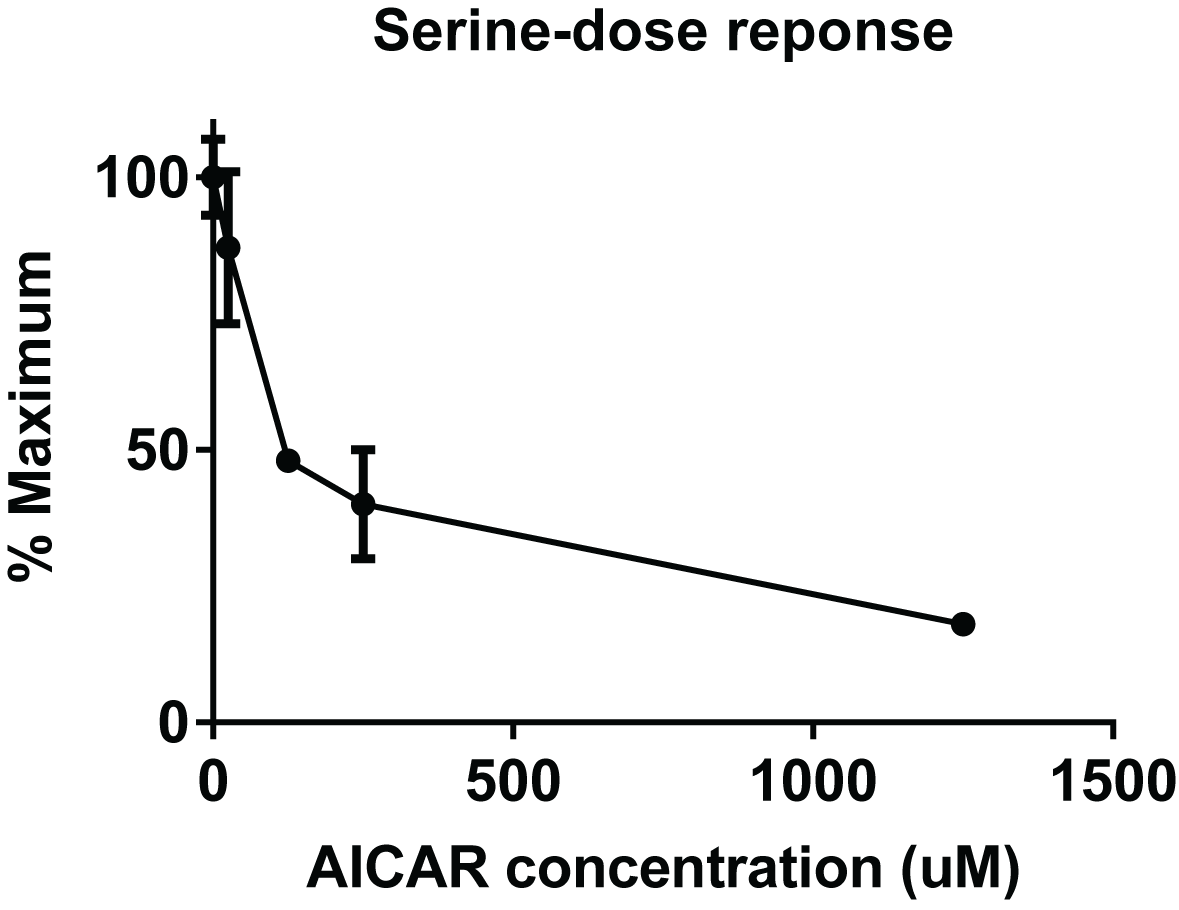

Supplement: S4 Fig — Levels of serine after increasing dose of AICAR (TIF) [file pone.0129029.s004.tif]
